# Tumour Necrosis Factor Alpha in Intestinal Homeostasis and Gut Related Diseases

**DOI:** 10.3390/ijms20081887

**Published:** 2019-04-16

**Authors:** Barbara Ruder, Raja Atreya, Christoph Becker

**Affiliations:** Department of Medicine 1, University of Erlangen-Nürnberg, Hartmannstr. 14, D-91052 Erlangen, Germany; Barbara.Ruder@uk-erlangen.de (B.R.); Raja.Atreya@uk-erlangen.de (R.A.)

**Keywords:** tumour necrosis factor, intestinal epithelium, IBD, cell death, anti-TNFα treatment, infectious disease

## Abstract

The intestinal epithelium constitutes an indispensable single-layered barrier to protect the body from invading pathogens, antigens or toxins. At the same time, beneficial nutrients and water have to be absorbed by the epithelium. To prevent development of intestinal inflammation or tumour formation, intestinal homeostasis has to be tightly controlled and therefore a strict balance between cell death and proliferation has to be maintained. The proinflammatory cytokine tumour necrosis factor alpha (TNFα) was shown to play a striking role for the regulation of this balance in the gut. Depending on the cellular conditions, on the one hand TNFα is able to mediate cell survival by activating NFκB signalling. On the other hand, TNFα might trigger cell death, in particular caspase-dependent apoptosis but also caspase-independent programmed necrosis. By regulating these cell death and survival mechanisms, TNFα exerts a variety of beneficial functions in the intestine. However, TNFα signalling is also supposed to play a critical role for the pathogenesis of inflammatory bowel disease (IBD), infectious diseases, intestinal wound healing and tumour formation. Here we review the literature about the physiological and pathophysiological role of TNFα signalling for the maintenance of intestinal homeostasis and the benefits and difficulties of anti-TNFα treatment during IBD.

## 1. Introduction

The Tumour Necrosis Factor (TNF) Superfamily is composed of 19 ligands and 29 receptors [1,2]. The transmembrane ligand proteins belonging to this family are all characterized by a conserved C-terminal domain, the so called TNF homology domain. In addition, the tumour necrosis factor receptors (TNFR) belonging to this family, share cysteine-rich extracellular domains, which bind to the TNF homology domains of the TNF ligands [3]. In general, TNF- and TNFR-mediated signalling pathways are involved in the regulation of cell survival, proliferation, morphogenetic changes and cell death [2]. Members of the Tumour Necrosis Factor Superfamily are expressed by many different cell types in the human body, for example, immune cells, hematopoietic cells, epithelial and endothelial cells under physiologic but also under pathologic conditions [2,4]. The name-giving term “Tumour Necrosis Factor” was first introduced by Carswell and colleagues in 1975 and named a factor which could induce necrosis in tumour cells [5]. Later, in the 1980s, two different types of TNF proteins were identified, which were called TNFα and TNFβ. These two TNF proteins showed more than 50% of sequence homology, however they differed in their molecular mass, were produced by different cell types and recognized by different antibodies [6,7,8,9,10].

The TNFα protein is produced by many different cell types in the human body, however the main producers are the cells from the monocytic lineage, for example, macrophages [11]. TNFα plays a pivotal role during steady state or pathologic conditions, for example, infections, injury, inflammation and tumour development [4,11,12]. Once released from macrophages, which constitute the first line of defence, TNFα activates other immune cells and mediates production of additional proinflammatory cytokines during inflammatory responses, therefore TNFα is mainly described to function as a proinflammatory cytokine [13,14]. TNFβ is expressed by T and B cells, Natural Killer cells and macrophages and plays fundamental roles for development and function of the immune system, for example, gut-associated lymphatic tissue [15,16], as well as during inflammation [17].

The TNFα protein is generated as a bioactive, 26 kDa transmembrane type 2 polypeptide precursor (mTNFα) [18]. Cleavage of mTNFα by TNFα-converting enzyme (TACE) results in release of a 17 kDa soluble TNFα (sTNFα), which can either act locally or enter the bloodstream, potentially acting far away from its production site [4,19]. mTNFα and sTNFα can both bind to two receptors, TNF receptor 1 and 2 (TNFR1 and 2), however, TNFR2 is mainly activated by mTNFα [20]. TNFR1 is ubiquitously expressed on several different cell types, whereas expression of TNFR2 is highly regulated by different stimuli and more restricted, for example, to certain immune cells, endothelial cells or neurons [21,22,23,24]. Therefore TNFR1 seems to be mediating TNFα signalling in most cell types. On the one hand, TNFR signalling can induce caspase-dependent apoptotic or caspase-independent necroptotic cell death respectively. On the other hand, activation of the two TNFRs can mediate cell survival via activation of the classical and alternative NFκB (Nuclear Factor kappa-light-chain-enhancer of activated B cells) as well as MAP Kinase pathways [25].

## 2. TNFα Is One of the Most Important Regulators of Cell Death and Survival

**Figure 1:**
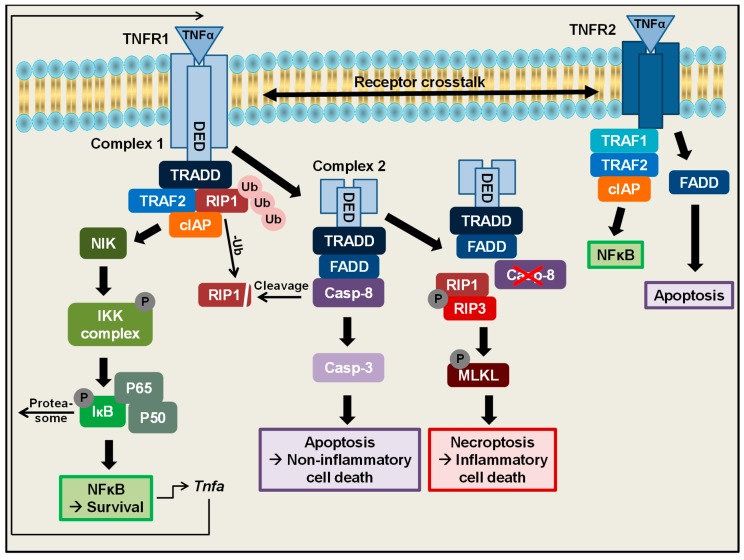
Regulation of cell death and survival by tumour necrosis factor alpha (TNFα). Binding of TNFα to TNFR1 leads to receptor trimerization and formation of receptor complex 1. This complex consists of TRADD, TRAF2, polyubiquitinylated RIP1 and cIAP and mediates cell survival via activation of NFκB. *TNFA* gene expression itself is regulated by NFκB. Depending on the cellular context, TNFα binding to TNFR1 can lead to formation of receptor complex 2, consisting of TRADD, FADD, deubiquitinylated RIP1 and Caspase-8. Activated caspase-8 cleaves RIP1 and further initiates a downstream caspase cascade to trigger apoptotic cell death, which is regarded as a non-inflammatory type of cell death. If caspase-8 activity is blocked, RIP1 cannot be cleaved anymore, leading to heterodimerization with RIP3 and autophosphorylation. Subsequently, RIP3 phosphorylates and activates MLKL, which oligomerizes, translocates to the cell membrane and initiates pore formation to induce necroptosis, which is described as a rather inflammatory type of cell death. In addition, depending on the cellular conditions binding of TNFα to TNFR2 can induce NFκB signalling or apoptosis, either independently or dependent on TNFR1 signalling via receptor crosstalk.

On a cellular level, one of the main functions of TNFα is the regulation of cell survival, which can be mediated by TNFR1 (Figure 1) [26]. TNFR1 is characterized by an intracellular C-terminal death domain, which is needed to deliver signals from the outer to the inner site of the cell [27]. As a first step in the TNFα-induced signalling cascade to mediate cell survival, binding of TNFα to TNFR1 leads to receptor trimerization [28], setting the base for the formation of a multimeric TNFR1 complex 1. Ligand binding and receptor trimerization are subsequently followed by association of the TRADD (TNFR-associated death domain) adapter protein with the cytoplasmic TNFR death domains [29]. Upon binding to TNFR1, TRADD further recruits other proteins to the TNFR1 complex: TRAF2 and polyubiquitinylated RIP1 [30,31,32]. TRAF2 in turn binds to cIAP1/2, which belong to the inhibitors of apoptosis proteins [33,34] and which are both stabilized in this TNF receptor complex by RIP1 [35]. This multimeric TNFR1 complex 1 mediates cell survival by activating the NFκB-inducing Kinase (NIK) leading to activation of the IκB kinase complex (IKK) [30,36,37]. The IKK complex subsequently phosphorylates the NFκB inhibitor IκB, leading to proteasomal degradation and unmasking of the nuclear localization signal of NFκB family proteins. This cascade finally enables the translocation of NFκB proteins to the nucleus to activate target gene expression of several genes, for example, *A1/Bfl1*, *Bcl-X_L_*, *cIAP* or *xIAP*, which exert antiapoptotic functions [38,39,40]. This signalling cascade altogether leads to activation of the NFκB signalling pathway to mediate cell survival. Interestingly the *Tnfa* promotor itself contains an NFκB binding site, leading to a positive autoregulation of its own gene expression [41]. Besides NFκB, TNFR 1 complex 1 activates different MAPK cascades including ERK, P38 MAP Kinase and JNK [42].

In addition to mediating cell survival and proliferation, TNFα is also described to be an important regulator of cell death (Figure 1). Depending on the cellular context, especially during stress conditions like inflammation or infection or lack of growth factors, cells are supposed to undergo a programmed cell death cascade, which finally culminates into caspase-dependent apoptosis. Under these apoptosis inducing conditions, an alternative complex, TNFR1 complex 2 can be formed. Binding of TNFα to TNFR1 under these conditions induces the dissociation of the intracellular TNFR1 death domains from the cellular membrane, followed by recruitment of additional proteins to this intracellular complex. Besides TNFR1, this complex 2 consists of TRADD, FADD [32] and further recruits caspase-8, as well as previously deubiquitinylated RIP1 [43]. In this complex, caspase-8 gets fully activated [44], subsequently cleaves RIP1 and finally triggers a cascade to activate the executioner caspase-3 [45]. These processes lead to induction of apoptotic cell death, which is characterized by membrane blebbing, cell shrinkage and nuclear disintegration, followed by the formation of apoptotic bodies, which can be engulfed by immune cells. Therefore apoptosis is considered to be a rather non-inflammatory type of cell death [43,46,47,48,49]. Interestingly, the activation status of caspase-8 in this TNFR complex can also be regulated by cellular FLIPs [50].

Interestingly, in 1988, Laster and colleagues observed that stimulation of certain cell types with TNFα does not only induce apoptotic cell death but also necrotic cell death, which is characterized by the lack of nuclear disintegration, the formation of a balloon-like plasma membrane and finally cell lysis [51]. Subsequent studies have shown that this kind of cell death usually occurs if the caspase-8 activity is blocked by genetic deletion or pharmacological inhibition [52,53,54,55,56,57,58]. Under these conditions, when caspase-8 activity is missing, TNFα binding to TNFR1 can induce another type of cell death, which in contrast to apoptosis occurs caspase-independently. Without Caspase-8 activity, RIP1 cannot be cleaved anymore, leading to the recruitment of another RIP kinase (RIP3) to the intracellular TNFR1 complex 2. Under these conditions the RIP kinases 1 and 3 form heterodimers, subsequently followed by rapid auto-phosphorylation and auto-activation of the latter [59]. Once RIP3 kinase is activated it phosphorylates and activates MLKL (Mixed lineage kinase domain like pseudokinase). To finally mediate TNFα-induced caspase-independent cell death, activated MLKL oligomerizes and translocates to the plasma membrane. There the MLKL oligomers are integrated into the phospholipid-bilayer and generate pores culminating in cell lysis [49,60,61]. This kind of cell death is termed classical necroptosis [62]. Necroptosis has recently gained a lot of interest and has been linked to the pathophysiology of several diseases including Inflammatory Bowel Disease (IBD) [63,64], however the precise mechanisms how necroptosis is regulated and executed in vivo remain to be fully elucidated. Of note, beside this classical RIP3-dependent necroptosis, another type of RIP3-independent programmed necrosis has been described in 2016 by our group [65].

Besides TNFR1-mediated regulation of cell death and survival, TNFα can also signal via TNFR2. This receptor, in contrast to TNFR1, does not contain any intracellular death receptor domain. However, under certain conditions, binding of TNFα to TNFR2 was also shown to induce NFκB signalling, which in this case is mediated by recruitment of TRAF2 and subsequently TRAF1, as well as cIAP1 and 2 to TNFR2 [66,67,68,69]. Moreover, also TNFR2-mediated activation of JNK has been described [70]. Of note, beside TNFR1 and TNFR2 signalling alone, under certain conditions also a crosstalk between these two TNF receptors has been revealed. In this crosstalk, TNFR2 was shown to induce degradation of TRAD2, leading to decreased NFκB activation and therefore an increased TNFR1-mediated cytotoxicity [71,72,73,74]. However, besides cell death regulation via crosstalk of the two TNF receptors, TNFR2 was additionally shown to mediate apoptotic death independently of TNFR1, suggesting that TNFR2 alone might also mediate both cell survival and cell death in a different way as compared to TNFR1 [75,76].

In summary, these studies underline that TNFα is a powerful pleiotropic cytokine which is able to regulate NFκB-mediated cell survival but also caspase-dependent and independent cell death via two different receptors and various pathways and protein complexes.

## 3. A Critical Role of TNFα for the Maintenance of Intestinal Homeostasis

The gastrointestinal tract is the biggest surface of the body with close contact to environmental factors. The intestine is lined with a single epithelial cell layer which separates the underlying tissue from the luminal contents. The intestinal lumen contains the microflora, consisting of fungi, viruses and bacteria, as well as nutrients, water and environmental toxins. The intestinal epithelial cell layer is characterized by an enormous self-renewing capacity (every 4-5 days), underlined by a tremendous turnover rate of the epithelial cells. In order to maintain the epithelial barrier, intestinal stem cells at the crypt bottom divide asymmetrically and give rise to new stem cells, as well as new daughter cells, which differentiate towards cells of the secretory and absorptive lineage along the crypt-villus axis. At the villus tips in the small intestine, differentiated epithelial cells are shed in the intestinal lumen. Only the secretory Paneth cells escape this migration towards the villus tip. Interestingly, they fully differentiate, remain at the crypts of Lieberkühn and survive for more than 3 weeks [77]. A strict balance between cellular proliferation at the crypt base and cell death at the villus tip must be preserved and is indispensable for maintaining intestinal homeostasis. Uncontrolled proliferation might culminate into tumour development, whereas increased cell death might lead to disintegration of the epithelial barrier followed by infiltration of bacteria and development of intestinal inflammation, like Crohn’s disease (CD) and ulcerative colitis (UC), which are the two main forms of IBD [78].

TNFα is mainly described as a proinflammatory cytokine which plays a critical role for maintaining the intestinal integrity but conversely also for the pathogenesis of intestinal inflammation. In the gut, beside immune cells, also intestinal epithelial cells, namely Paneth cells, have been shown to produce TNFα constitutively in mice as well as during inflammation in patients suffering from chronic inflammation [79,80]. Moreover, patients suffering from chronic intestinal inflammation show elevated levels of TNFα due to elevated numbers of TNFα secreting cells in the intestinal tissue [81,82,83,84]. In accordance, mice with a chronic overproduction of TNFα (*TNF^deltaARE^* mice) are characterized by spontaneous development of Crohn’s like inflammation in the small intestine [85]. Additionally, a variety of murine models of intestinal inflammation, for example, induction of DSS (Dextran Sulphate Sodium)- and TNBS (2,4,6-trinitrobenzenesulfonic acid)-mediated colitis or genetic models of spontaneous inflammation, such as in the *IL10^-/-^* or *Casp8^ΔIEC^* mouse models, are characterized by increased TNFα levels in the intestinal mucosa [52,86,87,88,89,90]. Moreover, in our own studies we observed increased *Tnfa* expression in the gut in a mouse model of chronic intestinal inflammation mediated by a single viral protein [91]. Interestingly, mice which are lacking the *Tnfa* gene were no longer susceptible to acute or chronic TNBS-induced colitis [88,89] whereas mice overexpressing *Tnfa* were more susceptible to chronic TNBS-induced colitis [88], suggesting a rather harmful function of TNFα signalling during TNBS-induced colitis. In contrast, reduced TNFα levels in mice mediated by anti-TNFα antibody treatment or genetic *Tnfa* knockout, aggravated DSS- induced colitis in acute models [89,92,93]. Conversely, during chronic DSS-induced colitis, anti-TNFα antibody treatment of mice reduced intestinal inflammation [92], implicating a beneficial effect of TNFα signalling during acute colitis but a rather harmful function during chronic colitis induced by DSS. In contrast to the two other models of experimentally induced colitis by DSS or TNBS, during oxazalone-induced colitis, *Tnfa* gene expression was not increased in colon samples as compared to controls. However, therapeutic administration of TNFα reduced colitis and histopathology [89]. These studies altogether implicate that TNFα might exert both beneficial as well as harmful functions in the gut depending on the inflammatory context. It is highly interesting that in the models of acute and chronic DSS-induced colitis, TNFα exerts different functions, despite the fact that the same chemical agent to induce inflammation in both models was used. One might speculate that during the acute DSS-induced colitis model, which mainly focuses on the effects of acute epithelial barrier disruption and innate immune reactions [94], TNFα might help to reduce intestinal barrier permeability and inflammation. In the model of chronic DSS-induced colitis, which particularly aims at investigating effects of a long-term inflammation and adaptive immune responses and which represents different stages of disease, including acute flares, wound healing and resolution [94], TNFα might rather act destructively in regard to inflammation and barrier integrity and therefore anti-TNFα treatment in this disease model might be beneficial.

**Figure 2:**
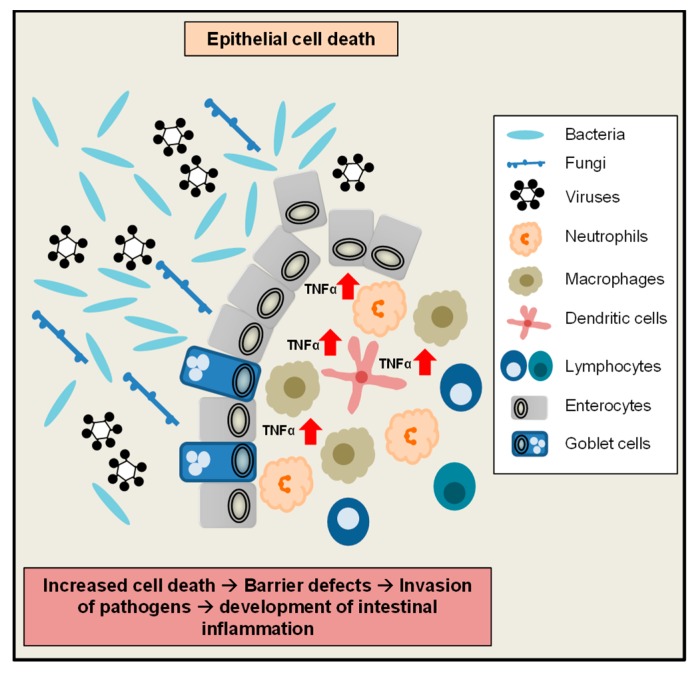
Regulation of epithelial cell death in the intestine by TNFα. Increased TNFα levels can trigger epithelial cell death in the intestine, which potentially leads to barrier defects and invasion of harmful pathogens. Altogether this might trigger or perpetuate the development of intestinal inflammation.

Under steady state conditions, during renewal of the intestinal epithelium, epithelial cells are shed into the lumen at the villus tip. Historically, intestinal cell shedding was considered to be a rather passive effect, where aged epithelial cells simply fall off the villus tip [95,96]. However, a variety of recent studies could show, that this process of epithelial shedding is highly regulated by tight junction protein complexes which rapidly close the gaps between enterocytes during physiologic shedding to preserve the intestinal barrier integrity [97,98,99]. Interestingly, elevated TNFα levels were shown to drive massive epithelial cell shedding (Figure 2) at the villus tip and to alter tight junction biology and intestinal permeability in vivo and in vitro [100,101,102,103,104]. Deregulated epithelial cell shedding and altered tight junction biology are features which are also seen in patients suffering from IBD [105,106,107]. However, whether the TNFα induced decrease in epithelial permeability is either mediated by TNFα-induced apoptosis or by deregulation of the tight junction biology is still a matter of debate [102,108,109,110]. Interestingly, mice lacking the apoptosis executioner caspase-3 do not show any morphological changes of the gut structure and colon length in comparison to controls [111], suggesting that epithelial turnover in the gut and cell shedding at least under steady state conditions are not mediated by caspase-3-triggered apoptosis. Of note, mice, which lacked functional NFκB signalling in IECs, were characterized by severe intestinal inflammation, accompanied by increased TNFα levels, massive amounts of dying apoptotic cells as well as infiltrating bacteria in the bowel wall. This phenotype was mediated by TNFR1-signaling [112], showing that increased TNFα levels might induce or contribute to barrier defects due to increased apoptosis, which constitute an entry point for invading harmful bacteria into the gut tissue to trigger or perpetuate intestinal inflammation. Taken together, these studies show that TNFα might play an important role for the regulation of apoptotic cell death and shedding in the intestine and a tight regulation of TNFα is important to maintain the epithelial barrier integrity.

Interestingly, in the gut it was shown that besides apoptosis, TNFα is also able to trigger caspase-independent necroptosis. Mice lacking caspase-8 in IECs (*Casp8^ΔIEC^*) were characterized by spontaneous development of ileitis accompanied by necroptotic RIP-dependent epithelial cell death and increased *Tnfa* gene expression, features which are also seen in patients suffering from Crohn’s disease. This spontaneous phenotype was dramatically worsened after additional TNFα administration in this model [52]. These results implicate that TNFα-induced necrotic cell death might contribute to the development of intestinal inflammation. However TNFα signalling alone was not sufficient to induce the spontaneous phenotype in *Casp8^ΔIEC^* mice, which was shown by the fact that additional deletion of TNFR1 in this model did not protect the animals from spontaneous inflammation and cell death [113]. Moreover, mice lacking FADD, an important adapter protein for TNFα-induced apoptotic cell death, in IECs, were also characterized by spontaneous development of inflammation in small and large intestine, accompanied by necroptotic epithelial cell death. Additional *Tnf* knockout could ameliorate but not completely abolish intestinal pathology in the colon, again suggesting that TNFα might contribute to development of intestinal inflammation. However, being in line with the study mentioned above, TNFα was not the main driver of inflammation and epithelial cell death in the small intestine [114], suggesting that cell death regulation in the small and large intestine differ. Taken together, these studies highlight that under pathogenic conditions like IBD, among other pathways, TNFα signalling might contribute to the development of intestinal inflammation, not only via induction of apoptosis but also necroptosis which is accompanied by development of inflammation.

**Figure 3:**
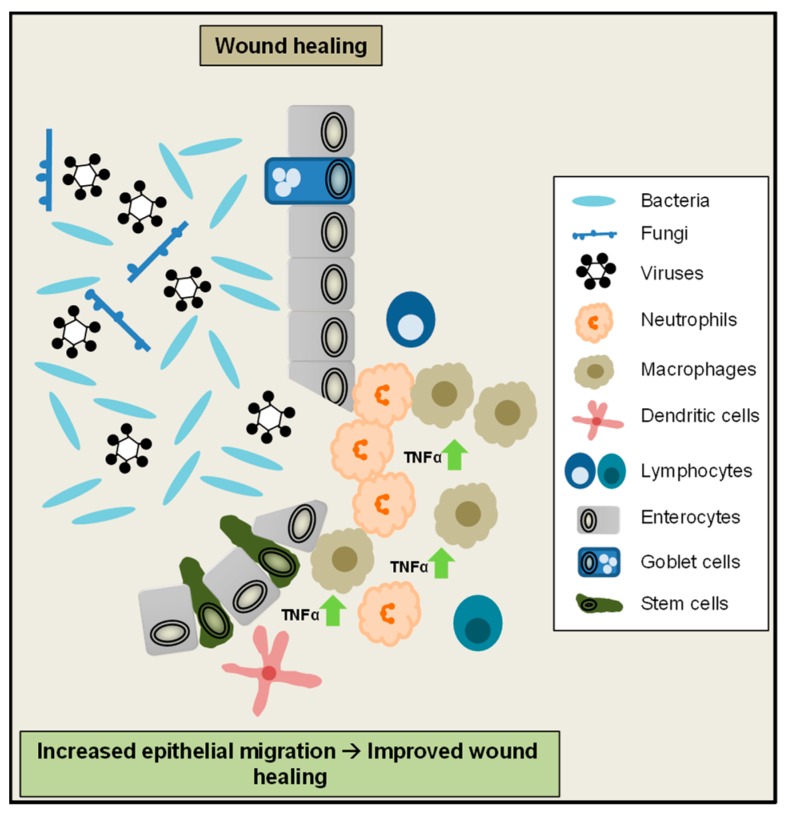
TNFα regulates intestinal cell survival and wound healing. Increased TNFα levels in the gut might drive epithelial cell survival and proliferation, leading to improved wound healing and mucosal repair after injury or during chronic colitis.

Beside epithelial cell shedding and cell death regulation, TNFα-mediated signalling also plays an important role for maintenance of the colonic epithelial barrier and wound healing. After disruption of the intestinal surface epithelium, during epithelial restitution, epithelial cells next to the injury form pseudopodia-like structures, migrate to the wound edge and cover the destroyed area. Afterwards, epithelial stem cells at the crypt base proliferate to compensate for cells lost during injury. To rebuild a functional cell layer at the injured site of the epithelium, these newly generated cells finally maturate and differentiate into the different cell types of the epithelium [115]. In an in vitro model of wound closure low amounts of TNFα promoted wound closure, whereas a higher dose failed to do so. Of note, TNFα-driven wound closure was mediated by TNFR2 in this model [116]. These observations are in line with a previous study, showing increased IEC proliferation due to low TNFα doses and reduced proliferation at higher doses [117]. A recent study moreover suggested an important role of epithelial TNFR signalling for mucosal repair in vivo during chronic colitis. In this setting, TNFα signalling promotes cell proliferation and wound repair via activation of epithelial Wnt/β-Catenin signalling, which is an important pathway to maintain the intestinal crypt-villus architecture [118,119,120]. Interestingly in IBD patients, as well as in the colon epithelium of mice exposed to DSS or injected with TNFα, growth factor receptor ErbB4 levels were increased. In an in vitro assay, decrease of ErbB4 levels diminished wound healing [121]. Therefore it was proposed that in the inflamed colonic epithelium, TNFα induced activation of ErbB4 promotes epithelial cell survival, wound healing and protects from ulceration during IBD [122]. Collectively these studies demonstrate that TNFα signalling plays an important role to promote epithelial cell migration and mucosal repair during intestinal inflammation (Figure 3).

**Figure 4:**
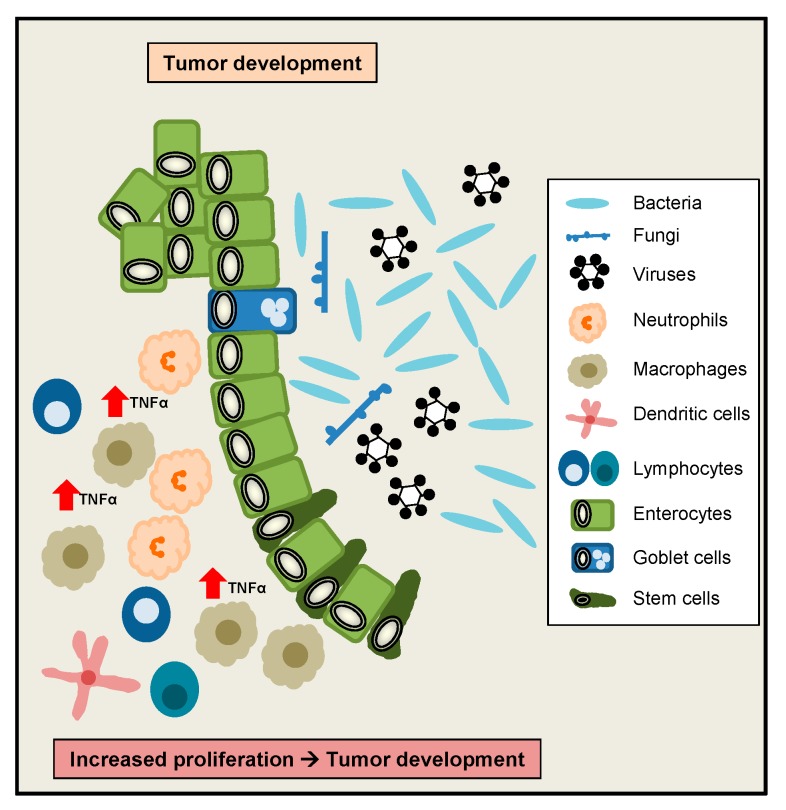
TNFα might induce intestinal tumour development. Elevated TNFα levels in the gut might contribute to colorectal cancer (CRC) and colitis-associated CRC development.

Interestingly, TNFα has initially been described as a factor released by host cells to induce tumour necrosis [5], therefore a role for TNFα to regulate tumour development and growth was implicated. As mentioned above, in vitro TNFα was shown to induce IEC proliferation at low doses, whereas at higher doses IEC proliferation was inhibited, implicating that high TNFα doses rather counteract proliferative effects [117]. However interestingly, in a colonoscopy-based cross-sectional study, circulating levels of TNFα were positively correlated with the occurrence of colorectal adenomas [123]. Moreover, significantly increased TNFα serum levels were observed in colorectal cancer (CRC) patients, with the highest levels in stage 4 CRC. *TNFA* gene expression was significantly higher in CRC as compared to adjacent normal colorectal tissues [124]. Additionally, patients with low TNFα serum levels were characterized by a higher survival rate as compared to patients with high TNFα serum levels [125]. Interestingly, another clinical study revealed that increased plasma levels of soluble TNFR2 are associated with an increased risk of CRC [126]. Beside these human studies, in a murine study a role for TNFα signalling during colitis-associated tumour development was suggested, since elevated levels of TNFα were found during colon carcinogenesis induced by AOM/DSS. Interestingly, mice lacking TNFR1 in this model developed less inflammation and fewer adenocarcinomatous lesions [127]. Taken together, despite its discovery as a tumour necrosis factor, elevated TNFα signalling might exert rather harmful functions during the development of CRC and colitis-associated CRC (Figure 4).

In summary, these studies show, that a critical regulation of TNFα levels seems to be a key player to maintain intestinal homeostasis. Under different conditions, TNFα might exert various beneficial but also harmful functions, again underlining its pleiotropic role for gut homeostasis.

## 4. Role of TNFα during Gastrointestinal Infection

**Figure 5:**
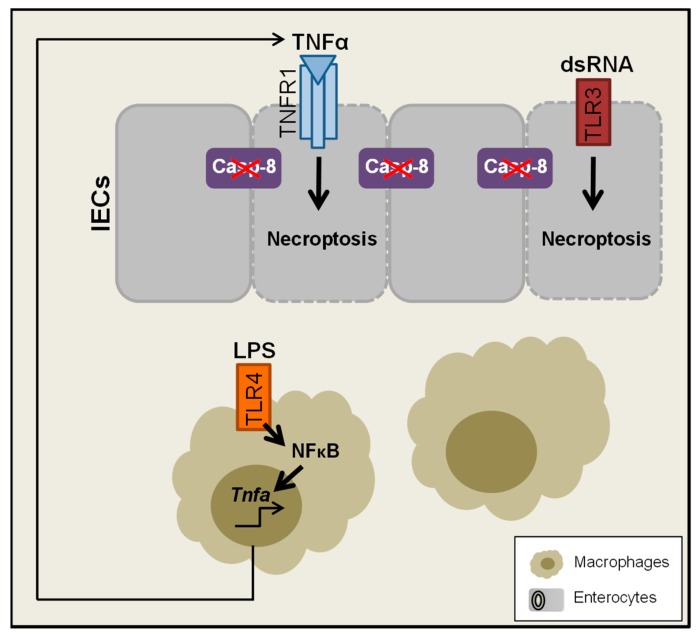
TNFα-dependent and independent TLR-mediated signalling in the gut. Administration of lipopolysaccharide (LPS) might activate TLR4 signalling in immune cells, to induce NFκB and subsequently gene expression of *Tnfa*, which in turn mediates TNFR1-driven epithelial cell death. dsRNA might directly bind to TLR3 on epithelial cells to trigger cell death, independently of TNFR1-signaling.

Interestingly in a recent study it was shown, that TNFα-driven intestinal inflammation in *TNF^deltaARE^* mice is fully dependent on the microbiota, since in germfree *TNF^deltaARE^* mice intestinal inflammation was completely absent. These data implicate an association between TNFα signalling, inflammation and the microbial composition [128]. In general increased TNFα levels are induced during inflammation or infection possibly perpetuating intestinal inflammation [11]. Mechanistically, in a murine model it was shown, that the bacterial cell wall compound Lipopolysaccharide (LPS) induced massive cell shedding, subsequently followed by villus shortening and diarrhoea. Of note, LPS-induced pathological shedding was strictly dependent on TNFR1 [110,129]. Furthermore in this model, LPS administration induced production of TNFα in intestinal immune cells, which in turn acts on epithelial cells, rather than directly activating TLR4-signaling in the epithelium (Figure 5) [129]. Altogether, these studies suggest, that during bacterial infection, increased TNFα signalling might exert rather harmful functions in intestinal epithelial cells.

Enteropathogenic and enterohemorrhagic *E.coli* strains (EPEC and EHEC) are known to induce diarrhoea and gastrointestinal disease worldwide especially in children [130]. One model to study the effects of an EHEC/EPEC infection in humans is the murine *Citrobacter rodentium* infection model. Interestingly, during *C. rodentium* infection in mice, TNFα is increased in the gut, however the absence of TNFα signalling due to *Tnfr* knockout in this model did not protect animals from infectious colitis. In contrast, the mice were even more susceptible to infectious colitis [131], suggesting a rather beneficial function on TNFα signalling during *C. rodentium* infection. Beside *C. rodentium*, also *Salmonella enterica* is a frequent cause of gastrointestinal infections in humans and animals worldwide every year. *Salmonella* Typhimurium infections lead to development of intestinal inflammation accompanied by diarrhoea, abdominal pain and vomiting [132]. Interestingly, infection of intestinal samples with *S*. Typhimurium ex vivo induced increased *Tnfa* gene expression levels. Moreover blocking of TNFα by administration of anti-TNFα antibodies prior to *S.* Typhimurium infection reduced gastrointestinal pathology, suggesting a rather harmful function of TNFα in the early phase of *Salmonella* infection [133]. Interestingly, in the more recently described murine model of *S*. Typhimurium induced colitis after streptomycin treatment, intestinal inflammation was also accompanied by increased levels of TNFα [134,135]. Another bacterium which is known to cause intestinal inflammation especially after antibiotic treatment is *Clostridium difficile*. *C. difficile* induced intestinal pathology ranges from mild diarrhoea to severe colitis [136]. Interestingly, patients suffering from *C. difficile* infection were characterized by elevated TNFα plasma and serum levels as compared to healthy controls [137,138]. In a murine model, infection with *C. difficile* also led to increased *Tnfa* gene expression in the colon of infected mice [139]. Moreover, murine macrophages were shown to secrete TNFα in response to stimulation with two different *C. difficile* toxins [140,141]. Interestingly, in the course of *C. difficile* infection, enhanced TNFα levels might be beneficial, since treatment of mice with anti-TNFα antibody shortly before *C. difficile* infection increased the inflammatory response and colonic histopathology [142].

Beside bacteria, also viruses mediate gastrointestinal pathology. for example Rotavirus is known to induce gastroenteritis worldwide, especially in children younger than 5 years [143]. Rotavirus infection was associated with increased TNFα levels in vitro [144,145,146] and children suffering from rotavirus induced diarrhoea were characterized by significantly increased TNFα serum levels as compared to healthy controls [147]. Interestingly, exogenous application of TNFα on Rotavirus-infected intestinal epithelial cells in vitro significantly reduced total viral RNA levels. In accordance, gene silencing of TNFR1 in these cells counteracted TNFα-mediated anti-Rotavirus effect [148], suggesting beneficial functions of TNFα signalling during Rotavirus infection.

As a response to infection, the host might activate cell death programs to limit further pathogen spreading. Many pathogens also evolutionary counteracted this induction of host cell death by expressing apoptosis inhibitors [149,150]. As it was shown by several studies, blocking of apoptosis especially by viral proteins potentially lead to induction of necroptotic, caspase-independent cell death [150,151,152,153,154,155]. In our own study, the impact of TNFR1 signalling to necroptotic cell death induced by bacteria or viruses (mimicked by LPS or viral dsRNA) was investigated. Interestingly only LPS-TLR4-induced necroptotic cell death was mediated by TNFR1-signaling, whereas viral dsRNA-TLR3-induced cell death could not be protected by *Tnfr1* knockout [129], suggesting a crucial role of TNF-signalling for TLR4-mediated bacteria-induced necroptotic cell death. We further suggested, that LPS-TLR4-signaling induced TNFα production in intestinal immune cells, further acting on epithelial cells via TNFR1, whereas dsRNA-TLR3 signalling rather directly mediated cell death in epithelial cells (Figure 5). Being in line, *S.* Typhimurium infected *Casp8^ΔIEC^* mice were characterized by reduced survival and an exacerbated course of infection as compared to controls and additional knockout of TNFR1 improved survival of infected mice [135].

## 5. Anti-TNFα Treatment Constitutes an Important Therapeutic Approach in IBD

CD and UC represent the major entities of IBD, which are defined as relapsing disorders of the gastrointestinal tract that are pathologically characterized by mucosal inflammation and epithelial damage not due to identifiable pathogens [156]. The clinical course is typically marked by unpredictable and progressive disease flares and clinical remission. Symptoms frequently comprise abdominal pain, chronic diarrhoea, sometimes severe bleeding, anaemia and fatigue. Moreover these systemic diseases are often accompanied by symptomatic extra-intestinal manifestations that can affect the joints, skin, eyes or other organs. These disease symptoms have a major impact on an individual’s quality of life and may also lead to various long-term structural complications, such as stenoses, fistulae, abscesses and heightened incidence of colitis-associated neoplasia [157,158]. There is therefore the urgent need for effective anti-inflammatory treatment in these patients. 20 years ago, the emergence, of monoclonal antibodies targeting TNFα revolutionized the treatment of IBD. The anti-TNFα agents infliximab (chimeric IgG1 monoclonal antibody), adalimumab (fully human IgG1 monoclonal antibody), certolizumab pegol (pegylated humanized Fab’ fragment monoclonal antibody) and golimumab (fully human IgG1 monoclonal antibody) have in the meanwhile been licensed for treatment of one or both IBD entities [159]. Furthermore, several biosimilars referencing infliximab or adalimumab have been approved for treatment of IBD patients [160]. The substance class of anti-TNFα agents is currently the most widely used biologic class in IBD therapy and is characterized by a fast onset of action and the ability to induce and maintain clinical response. Nevertheless, only subgroups of treated patients benefit from therapy, as over one-third of treated patients do not have a response following initiation of anti-TNFα therapy (primary non-response) and 30%–50% eventually lose response during the course of therapy (secondary non-response), resulting in exposure to potential side effects and toxicities without durable clinical benefit [159,161]. These findings highlight the dire clinical need for predictive biomarkers indicating anti-TNFα responsiveness in IBD patients. The precise mechanisms by which anti-TNFα agents convey their therapeutic efficacy in IBD remain rather elusive. As mere neutralization of sTNFα does not sufficiently explain its therapeutic property, recent data have indicated that several other mechanisms are critically involved in their mechanism of action in IBD. These include restoration of epithelial barrier function, Fcγ-receptor-mediated induction of wound healing macrophages, regulation of intestinal cell adhesion molecule expression, induction of antibody-dependent cellular cytotoxicity and complement-dependent cytotoxicity, induction of regulatory T cells, downregulation of mucosal angiogenesis and heightened migration of myofibroblasts [159]. In recent years, substantial amount of data has indicated that binding of TNFα antagonists to mTNFα and induction of mucosal T cell apoptosis are probably the most important factors that mediate the therapeutic efficacy of anti-TNFα therapy. The importance of mTNFα in IBD pathogenesis was demonstrated in experimental colitis models, where intestinal inflammation was induced upon transfer of naïve T-cells in mTNFα RAG2^-/-^ mice, which had a mutated TNFα that could not be cleaved into sTNFα [162]. Further data also indicated that specific neutralization of mTNFα but not sTNFα, was able to induce remission in T-cell-mediated colitis [163]. Fittingly, overexpression of TNFR2, the corresponding receptor to mTNFα signalling, in T-cells has been shown to aggravate experimental colitis [164] and the absence of TNFR2 attenuated spontaneous development of colitis in T cell receptor α knockout mice [165].

Several data in recent years have shown that expression of the target molecule in the inflamed tissue may affect response to anti-TNFα therapy in IBD. Our own studies have shown that anti-TNFα agents may bind to mTNFα expressing macrophages, thereby inducing apoptosis in TNFR2 expressing mucosal T cells lacking mTNFα-dependent co-stimulation. The mTNFα/TNFR2 signalling pathway is thus a crucial regulator in mediating resistance to apoptosis in intestinal T cells and may contribute to disease chronicity [166]. Indeed, apoptosis of mucosal T cells upon anti-TNFα therapy could be detected by ex vivo immunohistochemistry [166] and in vivo by molecular imaging in patients with active CD [167]. Apoptosis induction correlated with response to anti-TNFα therapy, whereas there was no evidence for heightened T cell apoptosis in non-responders. Using GMP-conform fluorescent labelled anti-TNFα antibodies for molecular in vivo imaging of mTNFα bearing mucosal cells in 25 CD patients, clinical response to subsequent anti-TNFα treatment could successfully be predicted. Patients with high amounts of mTNFα+ cells showed significantly higher short-term response rates at week 12 (92%) upon anti-TNFα therapy as compared to patients with low amounts of mTNFα+ cells (15%). This clinical response in the former patients was sustained over a follow-up period of one year and was associated with heightened incidence of mucosal healing [168]. Furthermore, other findings have similarly implicated that immune signalling pathways may predict and control responsiveness to anti-TNFα therapy in IBD [169]. This is best exemplified by recent studies where elevated pre-treatment expression of intestinal oncostatin-M [170] in mucosal plasma cells and inflammatory macrophages were associated with failure to anti-TNFα therapy [171]. These findings give hope for a personalized approach in anti-TNFα therapy in IBD. Here, it is equally important to understand the mechanisms underlying resistance to anti-TNFα therapy in IBD. A recent study indicates that the composition of intestinal immune cell infiltrates undergo marked changes under therapeutic pressure, leading to molecular resistance to anti-TNFα therapy in IBD. It was demonstrated that IL-23 derived by macrophages is one of the main drivers that evade anti-TNF-induced T cell apoptosis, leading to the expansion of apoptosis-resistant TNFR2+IL-23R+ T cells, which perpetuate the chronic intestinal inflammation in CD [172]. This IL-23-induced molecular resistance to anti-TNFα therapy in CD patients suggests that targeting IL-23 might be particularly effective in patients who fail anti-TNFα therapy. Indeed, recent studies have shown high response rates in anti-TNFα experienced CD patients using specific IL-23 p19 blockers [173,174].

Further understanding of the mechanism of action of anti-TNFα antibodies in IBD and the molecular resistance pathways that lead to non-response to TNFα inhibition will provide rational prediction of response, alternative molecular targets in no-responders and altogether a better therapeutic utilization of this substance class.

## 6. Conclusions

Taken together, the proinflammatory cytokine TNFα plays a fundamental role for the maintenance of intestinal homeostasis. It is believed to exert pivotal functions during physiological and pathophysiological conditions in the gut. Regulation of TNFα levels via anti-TNFα treatment is already an important therapeutic option for patients suffering from IBD. However, besides inflammation, several studies implicate a role of TNFα signalling during tumour development and also infectious diseases, therefore anti-TNFα treatment might be a starting point for additional therapeutic strategies to cure intestinal diseases.

## Abbreviations

IBD	Inflammatory Bowel Disease
CD	Crohn’s Disease
UC	Ulcerative Colitis
CRC	Colorectal cancer
IECs	Intestinal epithelial cells
TNFα	Tumour necrosis factor alpha
LPS	Lipopolysaccharide
mTNFα	Transmembrane TNFα
sTNFα	Soluble TNFα

## References

[1] Vanamee E.S., Faustman D.L. (2018). Structural principles of tumour necrosis factor superfamily signalling. Sci. Signal..

[2] Aggarwal B.B., Gupta S.C., Kim J.H. (2012). Historical perspectives on tumour necrosis factor and its superfamily: 25 years later, a golden journey. Blood.

[3] Bodmer J.L., Schneider P., Tschopp J. (2002). The molecular architecture of the TNF superfamily. Trends Biochem. Sci..

[4] Barbara J.A., Van ostade X., Lopez A. (1996). Tumour necrosis factor-alpha (TNF-alpha): The good, the bad and potentially very effective. Immunol. Cell Biol..

[5] Carswell E.A., Old L.J., Kassel R.L., Green S., Fiore N., Williamson B. (1975). An endotoxin-induced serum factor that causes necrosis of tumors. Proc. Natl. Acad. Sci. USA.

[6] Bringman T.S., Aggarwal B.B. (1987). Monoclonal antibodies to human tumour necrosis factors alpha and beta: Application for affinity purification, immunoassays, and as structural probes. Hybridoma.

[7] Kelker H.C., Oppenheim J.D., Stone-Wolff D., Henriksen-DeStefano D., Aggarwal B.B., Stevenson H.C., Vilcek J. (1985). Characterization of human tumour necrosis factor produced by peripheral blood monocytes and its separation from lymphotoxin. Int. J. Cancer.

[8] Aggarwal B.B., Henzel W.J., Moffat B., Kohr W.J., Harkins R.N. (1985). Primary structure of human lymphotoxin derived from 1788 lymphoblastoid cell line. J. Biol. Chem..

[9] Aggarwal B.B., Kohr W.J., Hass P.E., Moffat B., Spencer S.A., Henzel W.J., Bringman T.S., Nedwin G.E., Goeddel D.V., Harkins R.N. (1985). Human tumour necrosis factor. Production, purification, and characterization. J. Biol. Chem..

[10] Aggarwal B.B., Moffat B., Harkins R.N. (1984). Human lymphotoxin. Production by a lymphoblastoid cell line, purification, and initial characterization. J. Biol. Chem..

[11] Parameswaran N., Patial S. (2010). Tumour necrosis factor-alpha signalling in macrophages. Crit. Rev. Eukaryot. Gene Expr..

[12] Tracey K.J., Cerami A. (1994). Tumour necrosis factor: A pleiotropic cytokine and therapeutic target. Annu. Rev. Med..

[13] Maini R.N., Elliott M.J., Brennan F.M., Feldmann M. (1995). Beneficial effects of tumour necrosis factor-alpha (TNF-alpha) blockade in rheumatoid arthritis (RA). Clin. Exp. Immunol..

[14] Gamble J.R., Harlan J.M., Klebanoff S.J., Vadas M.A. (1985). Stimulation of the adherence of neutrophils to umbilical vein endothelium by human recombinant tumour necrosis factor. Proc. Natl. Acad. Sci. USA.

[15] De Togni P., Goellner J., Ruddle N.H., Streeter P.R., Fick A., Mariathasan S., Smith S.C., Carlson R., Shornick L.P., Strauss-Schoenberger J. (1994). Abnormal development of peripheral lymphoid organs in mice deficient in lymphotoxin. Science.

[16] Liepinsh D.J., Grivennikov S.I., Klarmann K.D., Lagarkova M.A., Drutskaya M.S., Lockett S.J., Tessarollo L., McAuliffe M., Keller J.R., Kuprash D.V. (2006). Novel lymphotoxin alpha (LTalpha) knockout mice with unperturbed tumour necrosis factor expression: Reassessing LTalpha biological functions. Mol. Cell Biol..

[17] Ware C.F. (2005). Network communications: Lymphotoxins, LIGHT, and TNF. Annu. Rev. Immunol..

[18] Kriegler M., Perez C., DeFay K., Albert I., Lu S.D. (1988). A novel form of TNF/cachectin is a cell surface cytotoxic transmembrane protein: Ramifications for the complex physiology of TNF. Cell.

[19] Sedger L.M., McDermott M.F. (2014). TNF and TNF-receptors: From mediators of cell death and inflammation to therapeutic giants—Past, present and future. Cytokine Growth Factor Rev..

[20] Grell M., Douni E., Wajant H., Lohden M., Clauss M., Maxeiner B., Georgopoulos S., Lesslauer W., Kollias G., Pfizenmaier K. (1995). The transmembrane form of tumour necrosis factor is the prime activating ligand of the 80 kDa tumour necrosis factor receptor. Cell.

[21] Puimege L., Libert C., Van Hauwermeiren F. (2014). Regulation and dysregulation of tumour necrosis factor receptor-1. Cytokine Growth Factor Rev..

[22] Faustman D.L., Davis M. (2013). TNF Receptor 2 and Disease: Autoimmunity and Regenerative Medicine. Front. Immunol..

[23] Luo D., Luo Y., He Y., Zhang H., Zhang R., Li X., Dobrucki W.L., Sinusas A.J., Sessa W.C., Min W. (2006). Differential functions of tumour necrosis factor receptor 1 and 2 signalling in ischemia-mediated arteriogenesis and angiogenesis. Am. J. Pathol..

[24] Vandenabeele P., Declercq W., Beyaert R., Fiers W. (1995). Two tumour necrosis factor receptors: Structure and function. Trends Cell Biol..

[25] Guicciardi M.E., Gores G.J. (2009). Life and death by death receptors. FASEB J..

[26] Tartaglia L.A., Rothe M., Hu Y.F., Goeddel D.V. (1993). Tumour necrosis factor’s cytotoxic activity is signaled by the p55 TNF receptor. Cell.

[27] Tartaglia L.A., Ayres T.M., Wong G.H., Goeddel D.V. (1993). A novel domain within the 55 kd TNF receptor signals cell death. Cell.

[28] Banner D.W., D’Arcy A., Janes W., Gentz R., Schoenfeld H.J., Broger C., Loetscher H., Lesslauer W. (1993). Crystal structure of the soluble human 55 kd TNF receptor-human TNF beta complex: Implications for TNF receptor activation. Cell.

[29] Hsu H., Xiong J., Goeddel D.V. (1995). The TNF receptor 1-associated protein TRADD signals cell death and NF-kappa B activation. Cell.

[30] Ea C.K., Deng L., Xia Z.P., Pineda G., Chen Z.J. (2006). Activation of IKK by TNFalpha requires site-specific ubiquitination of RIP1 and polyubiquitin binding by NEMO. Mol. Cell.

[31] Hsu H., Huang J., Shu H.B., Baichwal V., Goeddel D.V. (1996). TNF-dependent recruitment of the protein kinase RIP to the TNF receptor-1 signalling complex. Immunity.

[32] Hsu H., Shu H.B., Pan M.G., Goeddel D.V. (1996). TRADD-TRAF2 and TRADD-FADD interactions define two distinct TNF receptor 1 signal transduction pathways. Cell.

[33] Mahoney D.J., Cheung H.H., Mrad R.L., Plenchette S., Simard C., Enwere E., Arora V., Mak T.W., Lacasse E.C., Waring J. (2008). Both cIAP1 and cIAP2 regulate TNFalpha-mediated NF-kappaB activation. Proc. Natl. Acad. Sci. USA.

[34] Vince J.E., Pantaki D., Feltham R., Mace P.D., Cordier S.M., Schmukle A.C., Davidson A.J., Callus B.A., Wong W.W., Gentle I.E. (2009). TRAF2 must bind to cellular inhibitors of apoptosis for tumour necrosis factor (tnf) to efficiently activate nf-{kappa}b and to prevent tnf-induced apoptosis. J. Biol. Chem..

[35] Gentle I.E., Wong W.W., Evans J.M., Bankovacki A., Cook W.D., Khan N.R., Nachbur U., Rickard J., Anderton H., Moulin M. (2011). In TNF-stimulated cells, RIPK1 promotes cell survival by stabilizing TRAF2 and cIAP1, which limits induction of non-canonical NF-kappaB and activation of caspase-8. J. Biol. Chem..

[36] Malinin N.L., Boldin M.P., Kovalenko A.V., Wallach D. (1997). MAP3K-related kinase involved in NF-kappaB induction by TNF, CD95 and IL-1. Nature.

[37] Wu C.J., Conze D.B., Li T., Srinivasula S.M., Ashwell J.D. (2006). Sensing of Lys 63-linked polyubiquitination by NEMO is a key event in NF-kappaB activation [corrected]. Nat. Cell Biol..

[38] Brown K., Gerstberger S., Carlson L., Franzoso G., Siebenlist U. (1995). Control of I kappa B-alpha proteolysis by site-specific, signal-induced phosphorylation. Science.

[39] Palombella V.J., Rando O.J., Goldberg A.L., Maniatis T. (1994). The ubiquitin-proteasome pathway is required for processing the NF-kappa B1 precursor protein and the activation of NF-kappa B. Cell.

[40] Barkett M., Gilmore T.D. (1999). Control of apoptosis by Rel/NF-kappaB transcription factors. Oncogene.

[41] Baldwin A.S. (1996). The NF-kappa B and I kappa B proteins: New discoveries and insights. Annu. Rev. Immunol..

[42] Sabio G., Davis R.J. (2014). TNF and MAP kinase signalling pathways. Semin. Immunol..

[43] Micheau O., Tschopp J. (2003). Induction of TNF receptor I-mediated apoptosis via two sequential signaling complexes. Cell.

[44] Medema J.P., Scaffidi C., Kischkel F.C., Shevchenko A., Mann M., Krammer P.H., Peter M.E. (1997). FLICE is activated by association with the CD95 death-inducing signaling complex (DISC). EMBO J..

[45] Lin Y., Devin A., Rodriguez Y., Liu Z.G. (1999). Cleavage of the death domain kinase RIP by caspase-8 prompts TNF-induced apoptosis. Genes Dev..

[46] Ashkenazi A., Dixit V.M. (1998). Death receptors: Signaling and modulation. Science.

[47] Gunther C., Neumann H., Neurath M.F., Becker C. (2013). Apoptosis, necrosis and necroptosis: Cell death regulation in the intestinal epithelium. Gut.

[48] Lavrik I., Golks A., Krammer P.H. (2005). Death receptor signaling. J. Cell Sci..

[49] Zhang Y., Chen X., Gueydan C., Han J. (2018). Plasma membrane changes during programmed cell deaths. Cell Res..

[50] Irmler M., Thome M., Hahne M., Schneider P., Hofmann K., Steiner V., Bodmer J.L., Schroter M., Burns K., Mattmann C. (1997). Inhibition of death receptor signals by cellular FLIP. Nature.

[51] Laster S.M., Wood J.G., Gooding L.R. (1988). Tumor necrosis factor can induce both apoptic and necrotic forms of cell lysis. J. Immunol..

[52] Gunther C., Martini E., Wittkopf N., Amann K., Weigmann B., Neumann H., Waldner M.J., Hedrick S.M., Tenzer S., Neurath M.F. (2011). Caspase-8 regulates TNF-alpha-induced epithelial necroptosis and terminal ileitis. Nature.

[53] Holler N., Zaru R., Micheau O., Thome M., Attinger A., Valitutti S., Bodmer J.L., Schneider P., Seed B., Tschopp J. (2000). Fas triggers an alternative, caspase-8-independent cell death pathway using the kinase RIP as effector molecule. Nat. Immunol..

[54] Khwaja A., Tatton L. (1999). Resistance to the cytotoxic effects of tumor necrosis factor alpha can be overcome by inhibition of a FADD/caspase-dependent signaling pathway. J. Biol. Chem..

[55] Lin Y., Choksi S., Shen H.M., Yang Q.F., Hur G.M., Kim Y.S., Tran J.H., Nedospasov S.A., Liu Z.G. (2004). Tumor necrosis factor-induced nonapoptotic cell death requires receptor-interacting protein-mediated cellular reactive oxygen species accumulation. J. Biol. Chem..

[56] Ruemmele F.M., Dionne S., Levy E., Seidman E.G. (1999). TNFalpha-induced IEC-6 cell apoptosis requires activation of ICE caspases whereas complete inhibition of the caspase cascade leads to necrotic cell death. Biochem. Biophys. Res. Commun..

[57] Vercammen D., Beyaert R., Denecker G., Goossens V., Van Loo G., Declercq W., Grooten J., Fiers W., Vandenabeele P. (1998). Inhibition of caspases increases the sensitivity of L929 cells to necrosis mediated by tumor necrosis factor. J. Exp. Med..

[58] Wilson C.A., Browning J.L. (2002). Death of HT29 adenocarcinoma cells induced by TNF family receptor activation is caspase-independent and displays features of both apoptosis and necrosis. Cell Death Differ..

[59] Cho Y.S., Challa S., Moquin D., Genga R., Ray T.D., Guildford M., Chan F.K. (2009). Phosphorylation-driven assembly of the RIP1-RIP3 complex regulates programmed necrosis and virus-induced inflammation. Cell.

[60] Dondelinger Y., Declercq W., Montessuit S., Roelandt R., Goncalves A., Bruggeman I., Hulpiau P., Weber K., Sehon C.A., Marquis R.W. (2014). MLKL compromises plasma membrane integrity by binding to phosphatidylinositol phosphates. Cell Rep..

[61] Sun L., Wang H., Wang Z., He S., Chen S., Liao D., Wang L., Yan J., Liu W., Lei X. (2012). Mixed lineage kinase domain-like protein mediates necrosis signaling downstream of RIP3 kinase. Cell.

[62] Degterev A., Huang Z., Boyce M., Li Y., Jagtap P., Mizushima N., Cuny G.D., Mitchison T.J., Moskowitz M.A., Yuan J. (2005). Chemical inhibitor of nonapoptotic cell death with therapeutic potential for ischemic brain injury. Nat. Chem. Biol..

[63] Li S., Ning L.G., Lou X.H., Xu G.Q. (2018). Necroptosis in inflammatory bowel disease and other intestinal diseases. World J. Clin. Cases.

[64] Pierdomenico M., Negroni A., Stronati L., Vitali R., Prete E., Bertin J., Gough P.J., Aloi M., Cucchiara S. (2014). Necroptosis is active in children with inflammatory bowel disease and contributes to heighten intestinal inflammation. Am. J. Gastroenterol..

[65] Gunther C., He G.W., Kremer A.E., Murphy J.M., Petrie E.J., Amann K., Vandenabeele P., Linkermann A., Poremba C., Schleicher U. (2016). The pseudokinase MLKL mediates programmed hepatocellular necrosis independently of RIPK3 during hepatitis. J. Clin. Investig..

[66] Naude P.J., den Boer J.A., Luiten P.G., Eisel U.L. (2011). Tumor necrosis factor receptor cross-talk. FEBS J..

[67] Rothe M., Sarma V., Dixit V.M., Goeddel D.V. (1995). TRAF2-mediated activation of NF-kappa B by TNF receptor 2 and CD40. Science.

[68] Rothe M., Wong S.C., Henzel W.J., Goeddel D.V. (1994). A novel family of putative signal transducers associated with the cytoplasmic domain of the 75 kDa tumor necrosis factor receptor. Cell.

[69] Wang J., Ferreira R., Lu W., Farrow S., Downes K., Jermutus L., Minter R., Al-Lamki R.S., Pober J.S., Bradley J.R. (2018). TNFR2 ligation in human T regulatory cells enhances IL2-induced cell proliferation through the non-canonical NF-kappaB pathway. Sci. Rep..

[70] Cabal-Hierro L., Rodriguez M., Artime N., Iglesias J., Ugarte L., Prado M.A., Lazo P.S. (2014). TRAF-mediated modulation of NF-kB AND JNK activation by TNFR2. Cell Signal..

[71] Fotin-Mleczek M., Henkler F., Samel D., Reichwein M., Hausser A., Parmryd I., Scheurich P., Schmid J.A., Wajant H. (2002). Apoptotic crosstalk of TNF receptors: TNF-R2-induces depletion of TRAF2 and IAP proteins and accelerates TNF-R1-dependent activation of caspase-8. J. Cell Sci..

[72] Rodriguez M., Cabal-Hierro L., Carcedo M.T., Iglesias J.M., Artime N., Darnay B.G., Lazo P.S. (2011). NF-kappaB signal triggering and termination by tumor necrosis factor receptor 2. J. Biol. Chem..

[73] Siegmund D., Kums J., Ehrenschwender M., Wajant H. (2016). Activation of TNFR2 sensitizes macrophages for TNFR1-mediated necroptosis. Cell Death Dis..

[74] Weiss T., Grell M., Hessabi B., Bourteele S., Muller G., Scheurich P., Wajant H. (1997). Enhancement of TNF receptor p60-mediated cytotoxicity by TNF receptor p80: Requirement of the TNF receptor-associated factor-2 binding site. J. Immunol..

[75] Depuydt B., van Loo G., Vandenabeele P., Declercq W. (2005). Induction of apoptosis by TNF receptor 2 in a T-cell hybridoma is FADD dependent and blocked by caspase-8 inhibitors. J. Cell Sci..

[76] Vandenabeele P., Declercq W., Vanhaesebroeck B., Grooten J., Fiers W. (1995). Both TNF receptors are required for TNF-mediated induction of apoptosis in PC60 cells. J. Immunol..

[77] van der Flier L.G., Clevers H. (2009). Stem cells, self-renewal, and differentiation in the intestinal epithelium. Annu. Rev. Physiol..

[78] Atreya R., Neurath M.F. (2017). Current and Future Targets for Mucosal Healing in Inflammatory Bowel Disease. Visc. Med..

[79] Keshav S., Lawson L., Chung L.P., Stein M., Perry V.H., Gordon S. (1990). Tumor necrosis factor mRNA localized to Paneth cells of normal murine intestinal epithelium by in situ hybridization. J. Exp. Med..

[80] Lala S., Ogura Y., Osborne C., Hor S.Y., Bromfield A., Davies S., Ogunbiyi O., Nunez G., Keshav S. (2003). Crohn’s disease and the NOD2 gene: A role for paneth cells. Gastroenterology.

[81] Breese E.J., Michie C.A., Nicholls S.W., Murch S.H., Williams C.B., Domizio P., Walker-Smith J.A., MacDonald T.T. (1994). Tumor necrosis factor alpha-producing cells in the intestinal mucosa of children with inflammatory bowel disease. Gastroenterology.

[82] Dionne S., Hiscott J., D’Agata I., Duhaime A., Seidman E.G. (1997). Quantitative PCR analysis of TNF-alpha and IL-1 beta mRNA levels in pediatric IBD mucosal biopsies. Dig. Dis. Sci..

[83] Reimund J.M., Wittersheim C., Dumont S., Muller C.D., Kenney J.S., Baumann R., Poindron P., Duclos B. (1996). Increased production of tumour necrosis factor-alpha interleukin-1 beta, and interleukin-6 by morphologically normal intestinal biopsies from patients with Crohn’s disease. Gut.

[84] Reinecker H.C., Steffen M., Witthoeft T., Pflueger I., Schreiber S., MacDermott R.P., Raedler A. (1993). Enhanced secretion of tumour necrosis factor-alpha, IL-6, and IL-1 beta by isolated lamina propria mononuclear cells from patients with ulcerative colitis and Crohn’s disease. Clin. Exp. Immunol..

[85] Kontoyiannis D., Pasparakis M., Pizarro T.T., Cominelli F., Kollias G. (1999). Impaired on/off regulation of TNF biosynthesis in mice lacking TNF AU-rich elements: Implications for joint and gut-associated immunopathologies. Immunity.

[86] Arrieta M.C., Madsen K., Doyle J., Meddings J. (2009). Reducing small intestinal permeability attenuates colitis in the IL10 gene-deficient mouse. Gut.

[87] Berg D.J., Davidson N., Kuhn R., Muller W., Menon S., Holland G., Thompson-Snipes L., Leach M.W., Rennick D. (1996). Enterocolitis and colon cancer in interleukin-10-deficient mice are associated with aberrant cytokine production and CD4(+) TH1-like responses. J. Clin. Investig..

[88] Neurath M.F., Fuss I., Pasparakis M., Alexopoulou L., Haralambous S., Meyer zum Buschenfelde K.H., Strober W., Kollias G. (1997). Predominant pathogenic role of tumor necrosis factor in experimental colitis in mice. Eur. J. Immunol..

[89] Noti M., Corazza N., Mueller C., Berger B., Brunner T. (2010). TNF suppresses acute intestinal inflammation by inducing local glucocorticoid synthesis. J. Exp. Med..

[90] Yan Y., Kolachala V., Dalmasso G., Nguyen H., Laroui H., Sitaraman S.V., Merlin D. (2009). Temporal and spatial analysis of clinical and molecular parameters in dextran sodium sulfate induced colitis. PLoS ONE.

[91] Ruder B., Murtadak V., Sturzl M., Wirtz S., Distler U., Tenzer S., Mahapatro M., Greten F.R., Hu Y., Neurath M.F. (2018). Chronic intestinal inflammation in mice expressing viral Flip in epithelial cells. Mucosal Immunol..

[92] Kojouharoff G., Hans W., Obermeier F., Mannel D.N., Andus T., Scholmerich J., Gross V., Falk W. (1997). Neutralization of tumour necrosis factor (TNF) but not of IL-1 reduces inflammation in chronic dextran sulphate sodium-induced colitis in mice. Clin. Exp. Immunol..

[93] Naito Y., Takagi T., Handa O., Ishikawa T., Nakagawa S., Yamaguchi T., Yoshida N., Minami M., Kita M., Imanishi J. (2003). Enhanced intestinal inflammation induced by dextran sulfate sodium in tumor necrosis factor-alpha deficient mice. J. Gastroenterol. Hepatol..

[94] Wirtz S., Popp V., Kindermann M., Gerlach K., Weigmann B., Fichtner-Feigl S., Neurath M.F. (2017). Chemically induced mouse models of acute and chronic intestinal inflammation. Nat. Protoc..

[95] Leblond C.P. (1981). The life history of cells in renewing systems. Am. J. Anat..

[96] Leblond C.P., Stevens C.E. (1948). The constant renewal of the intestinal epithelium in the albino rat. Anat. Rec..

[97] Bullen T.F., Forrest S., Campbell F., Dodson A.R., Hershman M.J., Pritchard D.M., Turner J.R., Montrose M.H., Watson A.J. (2006). Characterization of epithelial cell shedding from human small intestine. Lab. Investug..

[98] Madara J.L. (1990). Maintenance of the macromolecular barrier at cell extrusion sites in intestinal epithelium: Physiological rearrangement of tight junctions. J. Membr. Biol..

[99] Marchiando A.M., Graham W.V., Turner J.R. (2010). Epithelial barriers in homeostasis and disease. Annu. Rev. Pathol..

[100] Al-Sadi R., Guo S., Ye D., Ma T.Y. (2013). TNF-alpha modulation of intestinal epithelial tight junction barrier is regulated by ERK1/2 activation of Elk-1. Am. J. Pathol..

[101] Ma T.Y., Boivin M.A., Ye D., Pedram A., Said H.M. (2005). Mechanism of TNF-{alpha} modulation of Caco-2 intestinal epithelial tight junction barrier: Role of myosin light-chain kinase protein expression. Am. J. Physiol. Gastrointest. Liver Physiol..

[102] Ma T.Y., Iwamoto G.K., Hoa N.T., Akotia V., Pedram A., Boivin M.A., Said H.M. (2004). TNF-alpha-induced increase in intestinal epithelial tight junction permeability requires NF-kappa B activation. Am. J. Physiol. Gastrointest. Liver Physiol..

[103] Marchiando A.M., Shen L., Graham W.V., Edelblum K.L., Duckworth C.A., Guan Y., Montrose M.H., Turner J.R., Watson A.J. (2011). The epithelial barrier is maintained by in vivo tight junction expansion during pathologic intestinal epithelial shedding. Gastroenterology.

[104] Marchiando A.M., Shen L., Graham W.V., Weber C.R., Schwarz B.T., Austin J.R., Raleigh D.R., Guan Y., Watson A.J., Montrose M.H. (2010). Caveolin-1-dependent occludin endocytosis is required for TNF-induced tight junction regulation in vivo. J. Cell Biol..

[105] Kiesslich R., Duckworth C.A., Moussata D., Gloeckner A., Lim L.G., Goetz M., Pritchard D.M., Galle P.R., Neurath M.F., Watson A.J. (2012). Local barrier dysfunction identified by confocal laser endomicroscopy predicts relapse in inflammatory bowel disease. Gut.

[106] Peeters M., Ghoos Y., Maes B., Hiele M., Geboes K., Vantrappen G., Rutgeerts P. (1994). Increased permeability of macroscopically normal small bowel in Crohn’s disease. Dig. Dis. Sci..

[107] Schmitz H., Barmeyer C., Fromm M., Runkel N., Foss H.D., Bentzel C.J., Riecken E.O., Schulzke J.D. (1999). Altered tight junction structure contributes to the impaired epithelial barrier function in ulcerative colitis. Gastroenterology.

[108] Gitter A.H., Bendfeldt K., Schulzke J.D., Fromm M. (2000). Leaks in the epithelial barrier caused by spontaneous and TNF-alpha-induced single-cell apoptosis. FASEB J..

[109] Schulzke J.D., Bojarski C., Zeissig S., Heller F., Gitter A.H., Fromm M. (2006). Disrupted barrier function through epithelial cell apoptosis. Ann. N. Y. Acad. Sci..

[110] Williams J.M., Duckworth C.A., Watson A.J., Frey M.R., Miguel J.C., Burkitt M.D., Sutton R., Hughes K.R., Hall L.J., Caamano J.H. (2013). A mouse model of pathological small intestinal epithelial cell apoptosis and shedding induced by systemic administration of lipopolysaccharide. Dis. Model. Mech..

[111] Brinkman B.M., Hildebrand F., Kubica M., Goosens D., Del Favero J., Declercq W., Raes J., Vandenabeele P. (2011). Caspase deficiency alters the murine gut microbiome. Cell Death Dis..

[112] Nenci A., Becker C., Wullaert A., Gareus R., van Loo G., Danese S., Huth M., Nikolaev A., Neufert C., Madison B. (2007). Epithelial NEMO links innate immunity to chronic intestinal inflammation. Nature.

[113] Wittkopf N., Gunther C., Martini E., He G., Amann K., He Y.W., Schuchmann M., Neurath M.F., Becker C. (2013). Cellular FLICE-like inhibitory protein secures intestinal epithelial cell survival and immune homeostasis by regulating caspase-8. Gastroenterology.

[114] Welz P.S., Wullaert A., Vlantis K., Kondylis V., Fernandez-Majada V., Ermolaeva M., Kirsch P., Sterner-Kock A., van Loo G., Pasparakis M. (2011). FADD prevents RIP3-mediated epithelial cell necrosis and chronic intestinal inflammation. Nature.

[115] Iizuka M., Konno S. (2011). Wound healing of intestinal epithelial cells. World J. Gastroenterol..

[116] Corredor J., Yan F., Shen C.C., Tong W., John S.K., Wilson G., Whitehead R., Polk D.B. (2003). Tumor necrosis factor regulates intestinal epithelial cell migration by receptor-dependent mechanisms. Am. J. Physiol. Cell Physiol..

[117] Kaiser G.C., Polk D.B. (1997). Tumor necrosis factor alpha regulates proliferation in a mouse intestinal cell line. Gastroenterology.

[118] Bradford E.M., Ryu S.H., Singh A.P., Lee G., Goretsky T., Sinh P., Williams D.B., Cloud A.L., Gounaris E., Patel V. (2017). Epithelial TNF Receptor Signaling Promotes Mucosal Repair in Inflammatory Bowel Disease. J. Immunol..

[119] Clevers H. (2006). Wnt/beta-catenin signaling in development and disease. Cell.

[120] Koch S., Nava P., Addis C., Kim W., Denning T.L., Li L., Parkos C.A., Nusrat A. (2011). The Wnt antagonist Dkk1 regulates intestinal epithelial homeostasis and wound repair. Gastroenterology.

[121] Frey M.R., Edelblum K.L., Mullane M.T., Liang D., Polk D.B. (2009). The ErbB4 growth factor receptor is required for colon epithelial cell survival in the presence of TNF. Gastroenterology.

[122] Hilliard V.C., Frey M.R., Dempsey P.J., Peek R.M., Polk D.B. (2011). TNF-alpha converting enzyme-mediated ErbB4 transactivation by TNF promotes colonic epithelial cell survival. Am. J. Physiol. Gastrointest. Liver Physiol..

[123] Kim S., Keku T.O., Martin C., Galanko J., Woosley J.T., Schroeder J.C., Satia J.A., Halabi S., Sandler R.S. (2008). Circulating levels of inflammatory cytokines and risk of colorectal adenomas. Cancer Res..

[124] Al Obeed O.A., Alkhayal K.A., Al Sheikh A., Zubaidi A.M., Vaali-Mohammed M.A., Boushey R., McKerrow J.H., Abdulla M.H. (2014). Increased expression of tumor necrosis factor-alpha is associated with advanced colorectal cancer stages. World J. Gastroenterol..

[125] Stanilov N., Miteva L., Dobreva Z., Stanilova S. (2014). Colorectal cancer severity and survival in correlation with tumour necrosis factor-alpha. Biotechnol. Biotechnol. Equip..

[126] Chan A.T., Ogino S., Giovannucci E.L., Fuchs C.S. (2011). Inflammatory markers are associated with risk of colorectal cancer and chemopreventive response to anti-inflammatory drugs. Gastroenterology.

[127] Popivanova B.K., Kitamura K., Wu Y., Kondo T., Kagaya T., Kaneko S., Oshima M., Fujii C., Mukaida N. (2008). Blocking TNF-alpha in mice reduces colorectal carcinogenesis associated with chronic colitis. J. Clin. Investig..

[128] Schaubeck M., Clavel T., Calasan J., Lagkouvardos I., Haange S.B., Jehmlich N., Basic M., Dupont A., Hornef M., von Bergen M. (2016). Dysbiotic gut microbiota causes transmissible Crohn’s disease-like ileitis independent of failure in antimicrobial defence. Gut.

[129] Gunther C., Buchen B., He G.W., Hornef M., Torow N., Neumann H., Wittkopf N., Martini E., Basic M., Bleich A. (2015). Caspase-8 controls the gut response to microbial challenges by Tnf-alpha-dependent and independent pathways. Gut.

[130] Robins-Browne R.M., Hartland E.L. (2002). Escherichia coli as a cause of diarrhea. J. Gastroenterol. Hepatol..

[131] Goncalves N.S., Ghaem-Maghami M., Monteleone G., Frankel G., Dougan G., Lewis D.J., Simmons C.P., MacDonald T.T. (2001). Critical role for tumor necrosis factor alpha in controlling the number of lumenal pathogenic bacteria and immunopathology in infectious colitis. Infect. Immun..

[132] Haraga A., Ohlson M.B., Miller S.I. (2008). Salmonellae interplay with host cells. Nat. Rev. Microbiol..

[133] Arnold J.W., Niesel D.W., Annable C.R., Hess C.B., Asuncion M., Cho Y.J., Peterson J.W., Klimpel G.R. (1993). Tumor necrosis factor-alpha mediates the early pathology in Salmonella infection of the gastrointestinal tract. Microb. Pathog..

[134] Barthel M., Hapfelmeier S., Quintanilla-Martinez L., Kremer M., Rohde M., Hogardt M., Pfeffer K., Russmann H., Hardt W.D. (2003). Pretreatment of mice with streptomycin provides a Salmonella enterica serovar Typhimurium colitis model that allows analysis of both pathogen and host. Infect. Immun..

[135] Hefele M., Stolzer I., Ruder B., He G.W., Mahapatro M., Wirtz S., Neurath M.F., Gunther C. (2018). Intestinal epithelial Caspase-8 signaling is essential to prevent necroptosis during Salmonella Typhimurium induced enteritis. Mucosal Immunol..

[136] Abt M.C., McKenney P.T., Pamer E.G. (2016). Clostridium difficile colitis: Pathogenesis and host defence. Nat. Rev. Microbiol..

[137] Czepiel J., Biesiada G., Brzozowski T., Ptak-Belowska A., Perucki W., Birczynska M., Jurczyszyn A., Strzalka M., Targosz A., Garlicki A. (2014). The role of local and systemic cytokines in patients infected with Clostridium difficile. J. Physiol. Pharmacol..

[138] Yu H., Chen K., Sun Y., Carter M., Garey K.W., Savidge T.C., Devaraj S., Tessier M.E., von Rosenvinge E.C., Kelly C.P. (2017). Cytokines Are Markers of the Clostridium difficile-Induced Inflammatory Response and Predict Disease Severity. Clin. Vaccine Immunol..

[139] Sadighi Akha A.A., Theriot C.M., Erb-Downward J.R., McDermott A.J., Falkowski N.R., Tyra H.M., Rutkowski D.T., Young V.B., Huffnagle G.B. (2013). Acute infection of mice with Clostridium difficile leads to eIF2alpha phosphorylation and pro-survival signalling as part of the mucosal inflammatory response. Immunology.

[140] Melo Filho A.A., Souza M.H., Lyerly D.M., Cunha F.Q., Lima A.A., Ribeiro R.A. (1997). Role of tumor necrosis factor and nitric oxide in the cytotoxic effects of Clostridium difficile toxin A and toxin B on macrophages. Toxicon.

[141] Sun X., He X., Tzipori S., Gerhard R., Feng H. (2009). Essential role of the glucosyltransferase activity in Clostridium difficile toxin-induced secretion of TNF-alpha by macrophages. Microb. Pathog..

[142] McDermott A.J., Higdon K.E., Muraglia R., Erb-Downward J.R., Falkowski N.R., McDonald R.A., Young V.B., Huffnagle G.B. (2015). The role of Gr-1(+) cells and tumour necrosis factor-alpha signalling during Clostridium difficile colitis in mice. Immunology.

[143] Tate J.E., Burton A.H., Boschi-Pinto C., Steele A.D., Duque J., Parashar U.D., Network W.H.-c.G.R.S. (2012). 2008 estimate of worldwide rotavirus-associated mortality in children younger than 5 years before the introduction of universal rotavirus vaccination programmes: A systematic review and meta-analysis. Lancet Infect. Dis..

[144] Deal E.M., Jaimes M.C., Crawford S.E., Estes M.K., Greenberg H.B. (2010). Rotavirus structural proteins and dsRNA are required for the human primary plasmacytoid dendritic cell IFNalpha response. PLoS Pathog.

[145] Mesa M.C., Rodriguez L.S., Franco M.A., Angel J. (2007). Interaction of rotavirus with human peripheral blood mononuclear cells: Plasmacytoid dendritic cells play a role in stimulating memory rotavirus specific T cells in vitro. Virology.

[146] Mohanty S.K., Ivantes C.A., Mourya R., Pacheco C., Bezerra J.A. (2010). Macrophages are targeted by rotavirus in experimental biliary atresia and induce neutrophil chemotaxis by Mip2/Cxcl2. Pediatr. Res..

[147] Azim T., Ahmad S.M., Sefat E.K., Sarker M.S., Unicomb L.E., De S., Hamadani J.D., Salam M.A., Wahed M.A., Albert M.J. (1999). Immune response of children who develop persistent diarrhea following rotavirus infection. Clin. Diagn. Lab. Immunol..

[148] Hakim M.S., Ding S., Chen S., Yin Y., Su J., van der Woude C.J., Fuhler G.M., Peppelenbosch M.P., Pan Q., Wang W. (2018). TNF-alpha exerts potent anti-rotavirus effects via the activation of classical NF-kappaB pathway. Virus Res..

[149] Faherty C.S., Maurelli A.T. (2008). Staying alive: Bacterial inhibition of apoptosis during infection. Trends Microbiol..

[150] Mocarski E.S., Upton J.W., Kaiser W.J. (2011). Viral infection and the evolution of caspase 8-regulated apoptotic and necrotic death pathways. Nat. Rev. Immunol..

[151] Huang Z., Wu S.Q., Liang Y., Zhou X., Chen W., Li L., Wu J., Zhuang Q., Chen C., Li J. (2015). RIP1/RIP3 binding to HSV-1 ICP6 initiates necroptosis to restrict virus propagation in mice. Cell Host Microbe.

[152] Kaiser W.J., Upton J.W., Mocarski E.S. (2013). Viral modulation of programmed necrosis. Curr. Opin. Virol..

[153] Maelfait J., Liverpool L., Bridgeman A., Ragan K.B., Upton J.W., Rehwinkel J. (2017). Sensing of viral and endogenous RNA by ZBP1/DAI induces necroptosis. EMBO J..

[154] Upton J.W., Kaiser W.J., Mocarski E.S. (2012). DAI/ZBP1/DLM-1 complexes with RIP3 to mediate virus-induced programmed necrosis that is targeted by murine cytomegalovirus vIRA. Cell Host Microbe.

[155] Wang X., Li Y., Liu S., Yu X., Li L., Shi C., He W., Li J., Xu L., Hu Z. (2014). Direct activation of RIP3/MLKL-dependent necrosis by herpes simplex virus 1 (HSV-1) protein ICP6 triggers host antiviral defense. Proc. Natl. Acad. Sci. USA.

[156] Atreya R., Neurath M.F. (2015). IBD pathogenesis in 2014: Molecular pathways controlling barrier function in IBD. Nat. Rev. Gastroenterol. Hepatol..

[157] Baumgart D.C., Sandborn W.J. (2012). Crohn’s disease. Lancet.

[158] Danese S., Fiocchi C. (2011). Ulcerative colitis. N. Engl. J. Med..

[159] Billmeier U., Dieterich W., Neurath M.F., Atreya R. (2016). Molecular mechanism of action of anti-tumor necrosis factor antibodies in inflammatory bowel diseases. World J. Gastroenterol..

[160] Radin M., Sciascia S., Roccatello D., Cuadrado M.J. (2017). Infliximab Biosimilars in the Treatment of Inflammatory Bowel Diseases: A Systematic Review. BioDrugs.

[161] Colombel J.F., Sandborn W.J., Reinisch W., Mantzaris G.J., Kornbluth A., Rachmilewitz D., Lichtiger S., D’Haens G., Diamond R.H., Broussard D.L. (2010). Infliximab, azathioprine, or combination therapy for Crohn’s disease. N. Engl. J. Med..

[162] Corazza N., Brunner T., Buri C., Rihs S., Imboden M.A., Seibold I., Mueller C. (2004). Transmembrane tumor necrosis factor is a potent inducer of colitis even in the absence of its secreted form. Gastroenterology.

[163] Perrier C., de Hertogh G., Cremer J., Vermeire S., Rutgeerts P., Van Assche G., Szymkowski D.E., Ceuppens J.L. (2013). Neutralization of membrane TNF but not soluble TNF, is crucial for the treatment of experimental colitis. Inflamm. Bowel Dis..

[164] Holtmann M.H., Douni E., Schutz M., Zeller G., Mudter J., Lehr H.A., Gerspach J., Scheurich P., Galle P.R., Kollias G. (2002). Tumor necrosis factor-receptor 2 is up-regulated on lamina propria T cells in Crohn’s disease and promotes experimental colitis in vivo. Eur. J. Immunol..

[165] Mizoguchi E., Mizoguchi A., Takedatsu H., Cario E., de Jong Y.P., Ooi C.J., Xavier R.J., Terhorst C., Podolsky D.K., Bhan A.K. (2002). Role of tumor necrosis factor receptor 2 (TNFR2) in colonic epithelial hyperplasia and chronic intestinal inflammation in mice. Gastroenterology.

[166] Atreya R., Zimmer M., Bartsch B., Waldner M.J., Atreya I., Neumann H., Hildner K., Hoffman A., Kiesslich R., Rink A.D. (2011). Antibodies against tumor necrosis factor (TNF) induce T-cell apoptosis in patients with inflammatory bowel diseases via TNF receptor 2 and intestinal CD14(+) macrophages. Gastroenterology.

[167] Van den Brande J.M., Koehler T.C., Zelinkova Z., Bennink R.J., te Velde A.A., ten Cate F.J., van Deventer S.J., Peppelenbosch M.P., Hommes D.W. (2007). Prediction of antitumour necrosis factor clinical efficacy by real-time visualisation of apoptosis in patients with Crohn’s disease. Gut.

[168] Atreya R., Neumann H., Neufert C., Waldner M.J., Billmeier U., Zopf Y., Willma M., App C., Munster T., Kessler H. (2014). In vivo imaging using fluorescent antibodies to tumor necrosis factor predicts therapeutic response in Crohn’s disease. Nat. Med..

[169] Atreya R., Neurath M.F. (2018). Mechanisms of molecular resistance and predictors of response to biological therapy in inflammatory bowel disease. Lancet Gastroenterol. Hepatol..

[170] West N.R., Hegazy A.N., Owens B.M.J., Bullers S.J., Linggi B., Buonocore S., Coccia M., Gortz D., This S., Stockenhuber K. (2017). Oncostatin M drives intestinal inflammation and predicts response to tumor necrosis factor-neutralizing therapy in patients with inflammatory bowel disease. Nat. Med..

[171] Gaujoux R., Starosvetsky E., Maimon N., Vallania F., Bar-Yoseph H., Pressman S., Weisshof R., Goren I., Rabinowitz K., Waterman M. (2018). Cell-centred meta-analysis reveals baseline predictors of anti-TNFalpha non-response in biopsy and blood of patients with IBD. Gut.

[172] Schmitt H., Billmeier U., Dieterich W., Rath T., Sonnewald S., Reid S., Hirschmann S., Hildner K., Waldner M.J., Mudter J. (2018). Expansion of IL-23 receptor bearing TNFR2+ T cells is associated with molecular resistance to anti-TNF therapy in Crohn’s disease. Gut.

[173] Feagan B.G., Sandborn W.J., D’Haens G., Panes J., Kaser A., Ferrante M., Louis E., Franchimont D., Dewit O., Seidler U. (2017). Induction therapy with the selective interleukin-23 inhibitor risankizumab in patients with moderate-to-severe Crohn’s disease: A randomised, double-blind, placebo-controlled phase 2 study. Lancet.

[174] Sands B.E., Chen J., Feagan B.G., Penney M., Rees W.A., Danese S., Higgins P.D.R., Newbold P., Faggioni R., Patra K. (2017). Efficacy and Safety of MEDI2070, an Antibody Against Interleukin 23, in Patients with Moderate to Severe Crohn’s Disease: A Phase 2a Study. Gastroenterology.

